# Supplementary material for: Comparative genomics provides new insights into the diversity, physiology, and sexuality of the only industrially exploited tremellomycete: Phaffia rhodozyma
Source: BMC Genomics. 2016 Nov 9;17:901. doi: 10.1186/s12864-016-3244-7 (PMC5103461; doi:10.1186/s12864-016-3244-7)
Supplement: Additional file 6: — List of orphan genes with links to PFAM (related to Additional file 1: Table S1). (ZIP 1428 kb) [file 12864_2016_3244_MOESM6_ESM.zip › BLAST_HTML_FTR/G00765_P.html]

BLAST Search Results


```
BLASTP 2.2.27+


Reference:
Stephen F. Altschul, Thomas L. Madden, Alejandro A. Schäffer,
Jinghui Zhang, Zheng Zhang, Webb Miller, and David J. Lipman (1997),
"Gapped BLAST and PSI-BLAST: a new generation of protein database
search programs", Nucleic Acids Res. 25:3389-3402.


Reference for
composition-based statistics:
Alejandro A. Schäffer, L. Aravind, Thomas L. Madden, Sergei
Shavirin, John L. Spouge, Yuri I. Wolf, Eugene V. Koonin, and
Stephen F. Altschul (2001), "Improving the accuracy of PSI-BLAST
protein database searches with composition-based statistics and
other refinements", Nucleic Acids Res. 29:2994-3005.


Database: nr
           71,551,133 sequences; 26,053,659,533 total letters


Query= G00765_P

Length=63
                                                                      Score     E
Sequences producing significant alignments:                          (Bits)  Value

emb|CED84576.1|  hypothetical protein [Xanthophyllomyces dendrorh...   130    1e-37
emb|CAP06086.1|  polyprotein, partial [Hepatitis C virus subtype 1b]  34.7    3.2  


 >emb|CED84576.1| hypothetical protein [Xanthophyllomyces dendrorhous]
Length=62

 Score =  130 bits (328),  Expect = 1e-37, Method: Compositional matrix adjust.
 Identities = 62/62 (100%), Positives = 62/62 (100%), Gaps = 0/62 (0%)

Query  1   MRVLESKTINEEGVTWGGASRKTLAIFSFLDPGHGRNVRAATVLQQHQRSQACVISDDAP  60
           MRVLESKTINEEGVTWGGASRKTLAIFSFLDPGHGRNVRAATVLQQHQRSQACVISDDAP
Sbjct  1   MRVLESKTINEEGVTWGGASRKTLAIFSFLDPGHGRNVRAATVLQQHQRSQACVISDDAP  60

Query  61  GD  62
           GD
Sbjct  61  GD  62


>emb|CAP06086.1| polyprotein, partial [Hepatitis C virus subtype 1b]
Length=177

 Score = 34.7 bits (78),  Expect = 3.2, Method: Compositional matrix adjust.
 Identities = 15/29 (52%), Positives = 20/29 (69%), Gaps = 0/29 (0%)

Query  11  EEGVTWGGASRKTLAIFSFLDPGHGRNVR  39
           E   TWG A+R TL I SFL+PG  +N++
Sbjct  57  ETHTTWGSAARTTLGIASFLNPGARQNIQ  85


Lambda      K        H        a         alpha
   0.326    0.137    0.422    0.792     4.96 

Gapped
Lambda      K        H        a         alpha    sigma
   0.267   0.0410    0.140     1.90     42.6     43.6 

Effective search space used: 659382356584


  Database: nr
    Posted date:  Sep 23, 2015 12:05 AM
  Number of letters in database: 26,053,659,533
  Number of sequences in database:  71,551,133


Matrix: BLOSUM62
Gap Penalties: Existence: 11, Extension: 1
Neighboring words threshold: 11
Window for multiple hits: 40
```
